# Evaluating the Correlation between Myofascial Pelvic Pain and Female Sexual Function: A Prospective Pilot Study

**DOI:** 10.3390/jcm13164604

**Published:** 2024-08-06

**Authors:** Lejla Sandrieser, Jana Heine, Christine Bekos, Alexandra Perricos-Hess, René Wenzl, Heinrich Husslein, Lorenz Kuessel

**Affiliations:** Department of Obstetrics and Gynecology, Medical University of Vienna, 1090 Vienna, Austria; lejla.sandrieser@meduniwien.ac.at (L.S.); jana.heine@meduniwien.ac.at (J.H.); christine.bekos@meduniwien.ac.at (C.B.); alexandra.perricos@meduniwien.ac.at (A.P.-H.); rene.wenzl@meduniwien.ac.at (R.W.); heinrich.husslein@meduniwien.ac.at (H.H.)

**Keywords:** sexual health, sexual dysfunction, dyspareunia, chronic pain, myofascial pain syndromes, trigger points

## Abstract

**Introduction:** Myofascial pelvic pain (MFPP) is a prevalent yet frequently overlooked condition characterized by myofascial trigger points located within the pelvic floor muscles. Women with MFPP often experience severely reduced quality of life due to impaired sexual health. Here, we examined the relationship between MFPP and sexual function. **Materials and Methods**: Eighty-three women with a benign gynecological condition were included in this pilot study. For each patient, we obtained a complete medical history, measured different types of subjective pain intensity using a visual analog scale, performed a validated standardized examination of the pelvic floor muscles for measuring MFPP, and used the German Female Sexual Function Index (FSFI-d) questionnaire. **Results**: Compared to women without MFPP (46 out of 83; 55.4%), the women with MFPP (37 out of 83; 44.6%) reported experiencing pain on more days per month (8 vs. 3 days/month; *p* = 0.002) and higher median VAS scores for dyspareunia (4 vs. 0; *p* < 0.001). We also found a significant inverse correlation between the severity of MFPP and overall FSFI-d scores (r = −0.35; *p* < 0.001), particularly in the FSFI-d subdomains of pain (r = −0.364; *p* < 0.001), lubrication (r = −0.230; *p* = 0.005), and arousal (r = −0.360; *p* < 0.001). **Conclusions:** Due to the higher prevalence of dyspareunia and pelvic pain, MFPP significantly impacts several aspects of female sexual health and function. This information, combined with increased awareness regarding MFPP, may provide a foundation for designing individualized therapies, thereby improving the quality of life of women affected by MFPP.

## 1. Introduction

Myofascial pain is a common yet underdiagnosed musculoskeletal disorder characterized by myofascial trigger points (MTrPs), which are small, palpable, hyperirritable nodules located on taut bands of skeletal muscle [1,2]. MTrPs typically occur in the muscles of the neck, the lower back, and the female pelvis [3,4,5], with a combined estimated lifetime prevalence of approximately 85% [1]. Although several theories have been suggested regarding the cause of MTrPs, their precise etiology remains unclear [6].

It is hypothesized that repeated episodes of microtrauma lead to continuous irritation of the surrounding tissue, thereby causing increased muscle tone and spasms in the affected muscles [1]. However, trauma does not appear to be a prerequisite for the development of MTrPs [7]. Even when muscle use exceeds the muscle’s capacity and normal recovery is disrupted, insufficient oxygenation or increased release of acetylcholine in the affected muscles may contribute to the formation of MTrPs [7]. In the long term, recurrent and persistent pain can subsequently lead to both peripheral and central sensitization manifesting as both motor and sensory impairments [8]. During this process, sustained stimulation of nociceptors causes increased sensitivity in the peripheral nerve fibers and in the neurons in the dorsal horn of the spinal cord, thereby lowering the response threshold, potentially leading to hyperalgesia and allodynia [9,10].

The viscerosomatic reflex involves chronic stimulation of nociceptors which originate from visceral organs being transmitted via afferent fibers to the dorsal horn, where the signal is then relayed to segmentally related somatic structures (e.g., muscles) via efferent fibers, eliciting reflective muscle tenderness [11]. Chronic muscle tension results in the development of MTrPs, which can be either spontaneously painful (i.e., active MTrPs), or painful only when the muscle is compressed (i.e., latent MTrPs) [2]. Once formed, MTrPs can persist as an autonomous source of ongoing peripheral nociceptive input and contribute to both peripheral and central sensitization [12,13,14]. Clinically, myofascial pain can present with similarities to various other chronic pain conditions, such as radiculopathy and pain stemming from the tendons or joints [15].

The pelvic region is a common location for myofascial pain. An estimated 13.2% of women experience myofascial pelvic pain (MFPP) [5], and an estimated 22–94% of patients with chronic pelvic pain present with symptoms characteristic of MFPP [3]. MFPP often occurs together with visceral disorders, such as interstitial cystitis and irritable bowel syndrome, as well as with chronic pain conditions, such as fibromyalgia and chronic fatigue syndrome [3,16]. Moreover, in the past decade, several studies have emphasized the high prevalence of MFPP among patients with endometriosis [2,17,18].

Chronic pain not only affects a woman’s physical state, but also reduces her quality of life, particularly with respect to her overall health, mental health, social functioning, and sexual health [19,20]. However, awareness regarding sexual dysfunction remains inadequate, and MFPP is often overlooked due to the lack of established guidelines for assessing MFPP [3,4,21]. Furthermore, strikingly little is known regarding the connection between MFPP and female sexual function.

Here, we examined the relationship between MFPP and female sexual health, using a standardized technique for assessing MFPP and the Female Sexual Function Index (FSFI) questionnaire as objective measurement tools.

## 2. Materials and Methods

### 2.1. Study Population and Interventions

Women who visited our outpatient clinic for ovarian cysts, uterine fibroids, or endometriosis were invited to participate in this prospective pilot study. We excluded the following groups: patients who were incapable of giving consent; patients with a confirmed diagnosis of HIV, tuberculosis, or hepatitis A, B, or C infection; patients with diagnosed malignancy; postmenopausal patients; and patients with a previously diagnosed cause of pelvic pain (e.g., a rheumatic disease such as systemic lupus erythematosus or ankylosing spondylitis), congenital pelvic deformity, orthopedic disorder affecting the spine or pelvis, chronic inflammatory bowel disease, or fibromyalgia. A total of 83 women 18–50 years of age who did not meet the exclusion criteria and agreed to participate were included in the study and provided their written informed consent. Basic demographic data such as the patient’s age, body mass index (BMI), gravidity, parity, medication history, and previous surgical procedures were recorded. All participants were asked to complete the German Female Sexual Function Index (FSFI-d) questionnaire [22]. The FSFI-d comprises six domains: desire, sexual arousal, lubrication, orgasm, sexual satisfaction, and pain. The questionnaire is rated on a scale from 2 to 36, with a total score of ≤26.55 indicating sexual dysfunction [22]. Furthermore, participating women were examined using the standardized internal palpation protocol reported by Meister et al. [23], with palpation starting at the center of the muscle belly, then along the length of the muscle, proceeding counterclockwise as follows: right obturator internus, right levator ani, left levator ani, and then left obturator internus; pain was reported on a scale ranging from 0 to 10. Pressure was standardized during all examinations by single-digit palpation of an area in the mid-thigh region. The sensation of pressure analogous to that sensed upon palpation of the mid-thigh was scored as 0. Any discomfort or pain beyond the sensation of pressure was assigned a score ranging from 1 to 10, with 1 corresponding to mild discomfort and 10 corresponding to the strongest imaginable pain. The scores of all examined sides were then added together [11]. As soon as the patient indicated a pain sensation scoring four on the visual analog scale in at least one palpation site, the patient was considered to have MFPP [23].

### 2.2. Statistics

Metric variables are presented as either the mean and standard deviation or the median and interquartile range. Nominal variables are presented as an absolute number and relative frequency. To compare baseline characteristics between the two groups, we used the non-parametric Mann–Whitney *U* test for metric variables, given the relatively small sample size; nominal variables were compared using the chi-square test. A correlation analysis was performed after testing for a normal distribution. To determine the association between the respective ordinal-scaled variables, we used the non-parametric Kendall’s Tau rank correlation coefficient. Differences with a two-tailed significance level of α = 0.05 were considered statistically significant. In the case of multiple independent tests, and to mitigate type I errors, we applied a Bonferroni correction; the newly adjusted significance level following Bonferroni correction was *p* < 0.008.

## 3. Results

In total, 83 women were included in this pilot study; baseline characteristics and pain characteristics are shown in Table 1.

**Figure 1:**
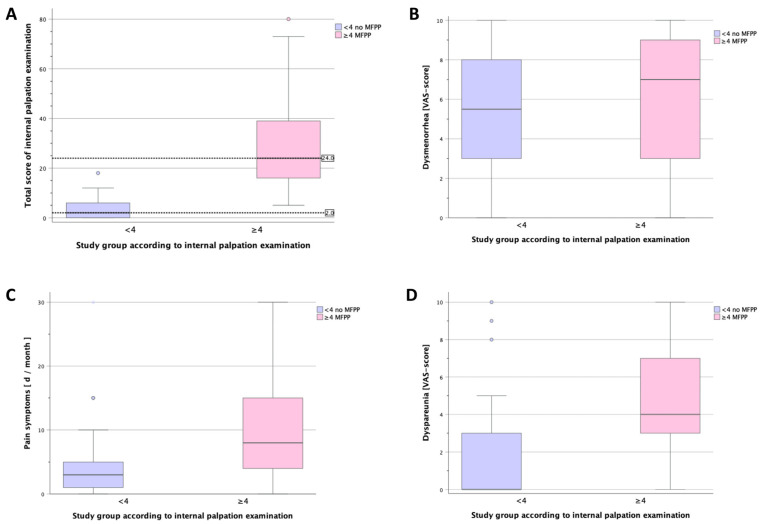
Comparison of internal palpation examination score (**A**), dysmenorrhea (**B**), pain symptoms (**C**), and dyspareunia (**D**) in women with and without MFPP. (**A**) Summary of internal palpation examination scores in the “no MFPP” and “MFPP” groups (*p* < 0.001). (**B**) Summary of mean dysmenorrhea intensity scores in the “no MFPP” and “MFPP” groups measured using a visual analog scale (*p* = 0.168). (**C**) Summary of the mean number of days per month with acyclic pain in the “no MFPP” and “MFPP” groups (*p* = 0.002). (**D**) Summary of the mean intensity of dyspareunia in the “no MFPP” and “MFPP” groups measured using a visual analog scale (*p* < 0.001).

Among these 83 participants, 37 (44.6%) presented with a pain score of ≥4 points in at least one palpation site and were classified as having MFPP (the “MFPP” group); the remaining 46 participants (55.4%) were classified as not having MFPP (the “no MFPP” group) (Figure 1A). We found that a significantly lower percentage of women with MFPP expressed a desire to have children compared to the women without MFPP (10.8% vs. 32.6%, respectively; *p* = 0.019). Furthermore, compared to women without MFPP, women with MFPP had a significantly higher median VAS score for dyspareunia (4 vs. 0; *p* < 0.001) (Figure 1D) and experienced acyclic pain on more days per month (8 days vs. 3 days; *p* = 0.002) (Figure 1C). We found no significant difference between groups with respect to the other parameters measured, including the median VAS score for dysmenorrhea (7 vs. 5.5; *p* = 0.168) (Figure 1B).

**Figure 2:**
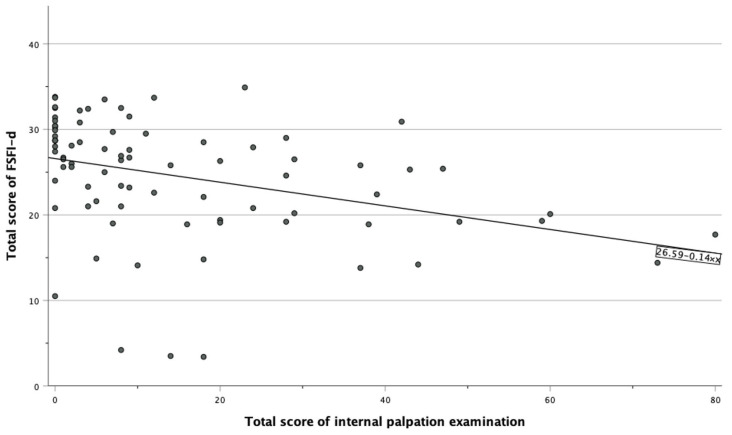
Total FSFI-d scores plotted against internal palpation examination scores for all 83 participants (r = −0.35; *p* < 0.001).

With respect to the relationship between MFPP and female sexual function, we found a significant inverse correlation between the severity of MFPP (i.e., the total score for the internal palpation examination) and sexual health (i.e., the total FSFI-d score) (r = −0.35; *p* < 0.001; Figure 2).

As shown in Table 2, after correcting for multiple testing, we also found a significant inverse correlation between MFPP scores and the FSFI-d subdomains of arousal, lubrication, and pain.

## 4. Discussion

Although myofascial pelvic pain (MFPP) significantly reduces both the quality of life and sexual health of women, it remains largely underdiagnosed and there are no standardized guidelines for its diagnosis or management [3,4,19,21]. Given that sexual health constitutes a fundamental human right [24], awareness regarding the link between MFPP and sexual dysfunction is imperative. Here, we analyzed the putative association between MFPP, measured using a standardized and validated internal palpation examination, and female sexual health, assessed using the validated FSFI-d questionnaire. This pilot study revealed a significant inverse correlation between the severity of MFPP and overall FSFI-d scores, and that MFPP is associated with significant changes in the FSFI-d subdomains of arousal, lubrication, and pain. Furthermore, we found that women with MFPP are significantly more likely to experience dyspareunia and/or acyclic pain compared to women without MFPP, with no difference observed regarding dysmenorrhea or dyschezia.

Our findings raise the question of whether the increased prevalence of dyspareunia among the women with MFPP may be attributed—at least in part—to stimulation of myofascial trigger points in the pelvic floor muscles due to deep penetration during sexual intercourse [2]. Additionally, we hypothesize that the increased frequency of acyclic pain reported by patients with MFPP may equally be attributed to the presence of myofascial trigger points, their deactivation and reactivation, and the underlying pain mechanism, which includes central and peripheral sensitization.

By serving as a potential underlying cause of dyspareunia and acyclic pain, MFPP may also explain why many patients with endometriosis-associated pain often do not receive adequate pain relief from hormone therapy or post-surgical interventions [25,26,27,28,29]. Indeed, studies that assessed the efficacy of hormone therapy for endometriosis-associated pelvic pain found that up to 19% of patients did not respond to treatment [29], with therapy-resistant acyclic pelvic pain often taking precedence [28]. Even after surgery for endometriosis, studies indicate that up to 38% of patients do not report pain reduction [25,26,27]. Given that neither hormonal nor surgical approaches affect the muscles, MFPP emerges as a plausible cause of pain in such cases. This notion is supported by a recent prospective cohort study by Orr et al. which associates persistent pain following endometriosis surgery with central sensitization [30]. We therefore believe that dyspareunia and acyclic pelvic pain may be attributed to MFPP, and we propose that standards for diagnosing all types of pelvic pain and assessing the efficiency of their respective therapies should include a standardized evaluation of MFPP.

Our findings on the FSFI-d subdomain of arousal are consistent with previous studies reporting that genito-pelvic pain is a known cause of arousal disorder [31]. It is also reasonable to infer that patients suffering from arousal disorder have difficulty with lubrication. On the other hand, it is interesting to note that, in our study, women with MFPP did not report a reduced ability to achieve orgasm, which may explain the lack of any significant difference between groups with respect to satisfaction. Nevertheless, given that previous studies showed significant changes in all six domains of the FSFI-d in patients with chronic pelvic pain [32,33], further studies are needed in order to better understand the extent and precise nature of the impaired sexual function in women with MFPP.

A strength of our study lies in our use of validated, reproducible methods for assessing MFPP and female sexual function. However, certain limitations may affect our interpretation of the results, including the limited sample size and the subcategorization method in which we used a cut-off value for inclusion in the MFPP group of ≥4 points in at least one palpation site during the internal palpation examination, based on the criteria reported by Meister et al. [23]. This relatively coarse division of patients into two groups (MFPP and no MFPP) resulted in high variations in pain intensity scores within the MFPP group, with total scores ranging from 4 to 80 points (see Figure 1). To date, only a limited number of studies have used this standardized examination method. Consequently, no unified guidelines exist for categorizing patients based on their level of pain, but such guidelines are needed in order to perform statistical analyses. Previous studies used modified methods to classify their study population into specific pain groups [34,35]. However, additional research is warranted in order to establish a standardized system for classifying pain intensity.

Importantly, sexual activity is affected by psychological, cultural, and social contexts, as well as various factors such as stress levels and relationship status [35]. However, these elements were not taken into account in our pilot study and should therefore be included in future research in order to achieve a more in-depth understanding of sexual health.

In conclusion, our results indicate that MFPP appears to play a fundamental role in dyspareunia and acyclic pain, and significantly affects female sexual health. Thus, increased awareness of MFPP, as well as standardized assessment of MFPP induced by MTrPs, may serve as a pivotal step toward providing comprehensive patient care, ultimately improving the quality of life of women affected by MFPP.

## Figures and Tables

**Table 1:** Baseline characteristics and pain characteristics.

Parameter	All Patients *n* = 83	No MFPP *n* = 46 (55.4%)	MFPP * n = 37 (44.6%)	*p*-Value
Age	32.7 (27.2–41.2)	32.9 (26.4–40.5)	31.7 (27.5–43.7)	0.700
Height [cm]	167 (163–170)	167 (163–170)	168 (163–172)	0.422
Weight [kg]	61 (55.5–77.0)	60.0 (55.8–76.5)	67 (55.0–80.0)	0.399
BMI [kg/m^2^]	22.66 (20.7–27.1)	21.9 (20.2–26.0)	22.96 (20.8–27.82)	0.618
Gravidity				
0	48 (57.8%)	29 (63.0%)	19 (51.4%)	0.666
1	15 (18.1%)	8 (17.4%)	7 (18.9%)	
2	9 (10.8%)	5 (10.9%)	4 (10.8%)	
3	3 (3.6%)	1 (2.2%)	2 (5.4%)	
4	6 (7.2%)	3 (6.5%)	3 (8.1%)	
5	2 (2.4%)	0	2 (5.4%)	
Paritys				
0	55 (66.3%)	30 (65.2%)	25 (67.6%)	0.799
1	13 (15.7%)	9 (19.6%)	4 (10.8%)	
2	10 (12.0%)	5 (10.9%)	5 (13.5%)	
3	3 (3.6%)	1 (2.2%)	2 (5.4%)	
4	2 (2.4%)	1 (2.2%)	1 (2.7%)	
Confirmed endometriosis				
yes	12 (14.5%)	8 (17.4%)	4 (10.8%)	0.397
no	71 (85.5%)	38 (82.6%)	33 (89.2%)	
Desire to have children				
yes	19 (22.9%)	15 (32.6%)	4 (10.8%)	0.019
no	64 (77.1%)	31 (67.4%)	33 (89.2%)	
Hormonal therapy				
No	62 (74.7%)	36 (78.3%)	26 (70.3%)	0.405
Yes	21 (25.3%)	10 (21.7%)	11 (29.7%)	
Bleeding	1 (1.2%)	0	1 (2.7%)	0.529
Pain	7 (8.4%)	3 (6.5%)	4 (10.8%)	
Pain + contraception	4 (4.8%)	1 (2.2%)	3 (8.1%)	
HRT	1 (1.2%)	0	1 (2.7%)	
Contraception	6 (7.2%)	4 (8.7%)	2 (5.4%)	
Other	2 (2.4%)	2 (4.3%)	0	
Menstrual cycle				
Amenorrhea	9 (10.8%)	4 (8.7%)	5 (13.5%)	0.508
Regular cycle	61 (73.5%)	33 (71.7%)	28 (75.7%)	
Irregular cycle	13 (15.7%)	9 (19.6%)	4 (10.8%)	
Pain				
Overall pain [days/month]	5.0 (2.0–15)	3 (1–5.5)	8 (4–15)	0.002
Dysmenorrhea [VAS]	7.0 (3.0–8.0)	5.5 (2.8–8.0)	7.0 (3.0–9.0)	0.168
Lower abdominal pain [VAS]	0 (0-0)	0 (0-0)	0 (0–4.0)	0.094
Dyspareunia [VAS]	3.0 (0–5.0)	0 (0–3.3)	4 (3.0–7.0)	<0.001
Dyschezia [VAS]	0 (0-0)	0 (0-0)	0 (0–3.0)	0.392
FSFI-d	26 (20.2–29.5)	28.3 (24.8–28.3)	20.8 (18.9–26.4)	0.089
Internal palpation	8 (1–23)	2 (0–6.5)	24 (15.0–40.5)	<0.001

* Defined as a pain score of ≥4 points in at least one of the palpation sites. For metric parameters, the median and interquartile range are presented. For ordinal/nominal parameters, the absolute number and percentage are presented. BMI—body mass index; VAS—visual analog scale; FSFI-d—German Female Sexual Function Index.

**Table 2:** Correlation analysis between FSFI-d subdomains and internal palpation examination scores, using Kendall’s Tau correlation, with corrected *p*-values shown.

	r	*p*-Value
FSFI-d subdomain		
Desire	−0.22	0.045
Arousal	−0.36	<0.001 *
Lubrication	−0.23	0.005 *
Orgasm	−0.18	0.027
Satisfaction	−0.18	0.028
Pain	−0.36	<0.001 *

* Significant after Bonferroni correction (*p* < 0.008). FSFI-d—German Female Sexual Function Index.

## Data Availability

Data supporting reported results are digitally stored and accessible to the study team. Upon request, the anonymized data can be made available.
